# Nosocomial infections in female compared with male patients with decompensated liver cirrhosis

**DOI:** 10.1038/s41598-022-07084-9

**Published:** 2022-02-28

**Authors:** Marie Griemsmann, Tammo L. Tergast, Nicolas Simon, Abdul-Rahman Kabbani, Michael P. Manns, Heiner Wedemeyer, Markus Cornberg, Benjamin Maasoumy

**Affiliations:** 1 Department of Gastroenterology, Hepatology and Endocrinology, Hannover Medical School, Carl-Neuberg-Strasse 1, 30625 Hannover, Germany; 2 Centre for Information Management (ZIMt), Hannover Medical School, Carl-Neuberg-Str.1, 30625 Hannover, Germany; 3 German Centre for Infection Research (Deutsches Zentrum Für Infektionsforschung DZIF), Partner-site Hannover-Braunschweig, Hannover, Germany; 4 Centre for Individualised Infection Medicine (CIIM), c/o CRC Hannover, Feodor-Lynen-Str. 15, 30625 Hannover, Germany

**Keywords:** Liver, Gastroenterology, Hepatology, Liver diseases, Liver cirrhosis

## Abstract

There are considerable differences between males and females regarding the etiology, progression and outcome of liver diseases. Infections are a frequent and severe complication in these patients. This study aimed to examine sex specific differences in the incidence and clinical course of nosocomial infections in patients with decompensated liver cirrhosis. A number of 556 consecutive hospitalized patients with decompensated liver cirrhosis and ascites were analyzed. The patients were followed up for the incidence of nosocomial infections, acute kidney injury (AKI), acute-on-chronic liver failure (ACLF) as well as liver transplantation and death (LTx-free survival). A number of 285 patients (111 women and 174 men) developed a nosocomial infection. Incidence was numerically lower in men (*P* = 0.076). While the frequency of a nosocomial spontaneous bacterial peritonitis was similar between males and females, the incidence of a nosocomial urinary tract infection was significantly higher in women (*P* < 0.001). No sex specific differences were documented regarding the outcome of an infection as indicated by a similar incidence of, AKI, ACLF as well as LTx-free survival. There seem to be no major differences in the incidence and outcome of nosocomial infections between male and female patients.

## Introduction

Over the recent years sex specific differences gain an increasing attention in medical research. Relevant differences include pharmacokinetic and pharmacodynamics of drugs, prevalence as well as natural history of several diseases^1–4^. This certainly also affects the field of hepatology. E.g. women are more likely to suffer from autoimmune hepatitis and primary biliary cholangitis^5,6^ whereas men develop more frequently primary sclerosing cholangitis^7^. Further, the progression of liver disease to fibrosis and cirrhosis differs. The toxic effect of alcohol is greater in females than in males, which leads to faster development of fibrosis in women with alcohol-related liver disease^8–10^. However, this progress is opposite in other etiologies of cirrhosis such as hepatitis B and C^11^, hemochromatosis and primary biliary cholangitis^9,12^. These differences were partly explained by diverse levels of sex hormones. Of note, the advantage of female sex in patients with hepatitis C diminishes after the menopause^9,13^.

Recently, several studies suggested sex specific differences in end stage liver disease. Female patients with decompensated liver cirrhosis on the waiting list for liver transplantation were more likely to die and less likely to be transplanted than male patients in some studies^14,15^. The impact of creatinine in MELD score has been suggested as a possible explanation^16,17^. However, this remains controversial. Of note, Mariante-Neto et al. did not observe any differences in survival and chance for transplant between sexes in a similar study design^18^ and the study by Umemura et al. even documented a better survival for female patients^19^.

One of the most common and severe complication in end stage liver disease are infections, particularly if acquired during hospitalization. The natural history of liver cirrhosis is significantly altered by an infection^20^. Mortality is four times increased^21^. Even after resolution of infection the survival remains impaired^20,22^. Moreover, infections trigger further cirrhosis-associated complications such as hepatic encephalopathy^23^, acute-on-chronic liver failure (ACLF)^24^ and acute kidney injury (AKI)^25^. Fast diagnosis and adequate treatment of infections as well as sufficient measures for prophylaxis are absolutely crucial, in particular in those with decompensated liver disease^26^. Therefore, it is of particular importance to know about the most common sites of infection and specific risk factors. Of note, the influence of sex on the incidence and clinical outcome of as well as risk factors for nosocomial infections in patients with decompensated liver cirrhosis has rarely been investigated in detail, so far.

This study aimed to study sex specific differences in incidence and the clinical course of as well as risk factors for nosocomial infections in a large real-world cohort of patients with decompensated liver cirrhosis and ascites.

## Patients and methods

### Patient cohort

A number of 1314 consecutive hospitalized patients who underwent a paracentesis from January 2012 until April 2018 at Hannover Medical School were considered for the study. In a first step, patients were selected automatically by the Enterprise Clinical Research Data Warehouse to avoid selection bias. Afterwards, the medical records of the patients were checked manually for inclusion and exclusion criteria leaving 556 individuals for the final analysis. Exclusion criteria included insufficient evidence of cirrhosis, malignant tumor disease other than hepatocellular carcinoma within the MILAN criteria, secondary intraabdominal infection, no sufficient follow up of nosocomial infection, HIV infection, congenital immune dysfunction, history of organ transplantation and no sufficient informed consent (Suppl. Fig. 1).

### Data collection

Clinical information like presence of infection, age, medication, AKI, ACLF and death was collected manually from the patients’ files. Laboratory values at time of admission and time of infection were extracted automatically by the Enterprise Clinical Research Data Warehouse. Diagnosis of liver cirrhosis was confirmed by ultrasound, elastography, liver histology, biochemical results and/or a combination of the above. Definition of infection was based on the judgement of the treating physician and/or clinical symptoms in combination with the following criteria:

- Spontaneous bacterial peritonitis (SBP): ≥ 500 nucleus containing cells/mm^3^ ascites fluid
- Urinary tract infection (UTI): leukocyturia and/or positive urine cultures and/or significant germination number as well as respective clinical signs of infection
- Pneumonia: evidence of pulmonary infiltrates in X-ray and respective clinical signs of infection
- Blood stream infection: clinical signs of infection and positive blood cultures

AKI and ACLF were defined according to the guidelines of “The European Association for the Study of the Liver” (EASL)^24,27–30^.

### Study design

#### Sex specific differences in the incidence of nosocomial infections

The patients were followed up 28 days from hospital admission for nosocomial infections. A competing risk analysis was performed handling death and LTx as competing events. As potential risk factors for any nosocomial infection sex, diabetes, age as well as MELD score, and platelets (both indicating the severity of liver disease) were included. In the univariate and multivariate competing risk analysis for incidence of nosocomial SBP the following parameters were considered for the model: presence of peritoneal catheter, antibiotic prophylaxis with norfloxacin and prior SBP. When analyzing potential risk factors for nosocomial UTI presence of a urinary catheter was added to the multivariate model. In a second step the cohort was divided into the two subgroups (female and male patients) to evaluate if specific risk factors for the development of a nosocomial infection are sex-specific.

### Sex specific differences in the outcome of nosocomial infection

#### LTx-free survival

Primary end point was liver transplantation or death (LTx-free survival) within 28 days after the onset of any nosocomial infection, SBP and UTI, respectively. Parameters considered for the univariate and multivariate Cox-Regression model were:

- Patients characteristics: sex, age, diabetes and esophageal varices
- Medication: β-Blocker and PPI intake
- Laboratory values: MELD score, albumin, CRP, leukocytes, sodium and platelets. Protein in ascites and nucleus containing cells in ascites were additionally included in the analysis for LTx-free survival after SBP.

#### Incidence of AKI

A competing risk model with LTx and death as competing risk was chosen. Follow-up was 28-days after the onset of nosocomial infection. All patients with dialysis or occurrence of AKI before the onset of nosocomial infection were excluded from this analysis. The multivariate competing risk model was adjusted to the parameters sex, MELD score, CRP and platelets. All laboratory values were assessed at the onset of the infection.

#### Incidence of ACLF

A competing risk analysis with death and LTx as competing risk was performed. Follow-up was 28-days after the onset of nosocomial infection. The multivariate competing risk model was adjusted to the parameters sex, MELD score, CRP and platelets.

### Statistics

All analyses were performed with IBM SPSS Statistics 26. Continuous variables were expressed as means and categorical variables as percentages. T-test was used for continuous variables and chi-square-test for categorical variables.

To asses LTx-free survival univariate and multivariate Cox-regression (backward conditional) were applied. All parameters with *P* ≤ 0.05 and the investigated factor sex were included in multivariate analysis.

Incidence of nosocomial infection, incidence of AKI and incidence of ACLF were analyzed by competing risk analysis. Multivariate competing risk analyses were performed in R Studio 3.5.2 with ‘crrstep-package’^31–33^. Backward direction was chosen and AIC criterion selected. Transformation into p-values and Hazard Ratios was done by using the function crrstep.output^34^. Cumulative incidences were executed with ‘cmprsk’ package^35^.

### Ethics

The study was approved by the local ethic committee of Hannover Medical School (Nr. 7935_BO_K_2018) and was performed according to the Declaration of Helsinki. All included patients provided sufficient written informed consent for the scientific use of their clinical data at hospital admission.

## Results

### Study cohort

Overall, 203 (36.5%) women and 353 (63.5%) men were included in the study cohort. The mean age of the individuals was 57 years. There were some differences in the baseline characteristics between female and male patients: Alcohol-related liver disease was more common in men (*P* = 0.002) whereas cholestatic (*P* = 0.011) etiology was more frequent in women. Esophageal varices were more often present in male than in female patients (*P* = 0.003). Further, the MELD score (*P* = 0.016) and the Serum-Creatinine (*P* = 0.003) were higher in men. Moreover, men had a lower platelet count (*P* = 0.029). The detailed baseline characteristics are displayed in Table 1.

**Table 1:** Baseline characteristics.

Variable	All patients	Female	Male	*P*-value
		(n = 203, 36.5%)	(n = 353, 63.5%)	
Age (years)	56.8 (11.1)	56.3 (11.4)	57.1 (11.0)	0.448
**Etiology of liver cirrhosis**				
Alcohol-related	52% (n = 291)	44% (n = 89)	57% (n = 202)	**0.002**
Cryptogenic	11% (n = 61)	13% (n = 27)	10% (n = 34)	0.183
Viral	18% (n = 102)	17.2% (n = 35)	19% (n = 67)	0.61
NASH	7% (n = 38)	7% (n = 15)	7% (n = 23)	0.694
Cholestatic	8% (n = 42)	11% (n = 23)	5% (n = 19)	**0.011**
Other	14% (n = 79)	16% (n = 33)	13% (n = 46)	0.294
Mixed	10% (n = 55)	9% (n = 19)	10% (n = 36)	0.75
**Cause of hospitalization**				
Ascites	65% (n = 361)	65% (n = 131)	65% (n = 230)	0.882
TIPS-Evaluation	20% (n = 110)	16% (n = 33)	22% (n = 77)	0.113
LTx-Evaluation	14%(n = 78)	14% (n = 29)	14% (n = 49)	0.895
HE	6% (n = 32)	5% (n = 10)	6% (n = 22)	0.524
HRS	3% (n = 17)	1% (n = 3)	4% (n = 14)	0.101
Worsening of general condition	5% (n = 26)	4% (n = 9)	5% (n = 17)	0.837
Infection	2% (n = 10)	2% (n = 4)	2% (n = 6)	0.817
ACLF	2% (n = 10)	2% (n = 4)	2% (n = 6)	0.817
Other cause	29%(n = 161)	34% (n = 69)	26% (n = 92)	** 0.047**
PPI intake	81% (n = 445)	77% (n = 154)	83% (n = 291)	0.078
Β-blockers	38% (n = 212)	34% (n = 69)	41% (n = 143)	0.136
Esophageal varices	76% (n = 422)	69% (n = 140)	80% (n = 282)	**0.003**
Diabetes	24% (n = 135)	23% (n = 47)	25% (n = 88)	0.638
MELD score	18 (7.0)	17 (6.6)	19 (7.2)	**0.016**
Albumin (g/l)	27.1 (5.9)	26.6 (5.4)	27.4 (6.2)	0.273
Bilirubin (µmol/l)	91.9 (135.4)	86.6 (130.6)	94.9 (138.2)	0.488
CRP (mg/l)	30.4 (34.7)	30.1 (38.3)	30.5 (32.5)	0.903
INR (Ratio)	1.53 (0.46)	1.49 (0.37)	1.55 (0.50)	0.209
Creatinine (µmol/l)	130.6 (89.2)	115.6 (70.5)	139.2 (97.3)	**0.003**
Leukocytes (10^3^/µl)	8.8 (6.1)	9.3 (5.9)	8.6 (6.2)	0.164
Platelets (10^3^/µl)	147.6 (102.7)	160 (119.7)	140 (90.8)	**0.029**
Sodium (mmol/l)	134 (5.4)	134 (5.5)	134 (5.3)	0.863
Antibiotic intake within 12 weeks before admission	33% (n = 180)	32% (n = 64)	33% (n = 116)	0.818

All continues variables are displayed as mean with standard deviation. Categorical variables are displayed as proportions.

Laboratory values were assessed at time of admission.

In some patients several causes of hospitalization were present.

Significant values are in bold.

*MELD* model of end stage liver disease, *HE* hepatic encephalopathy, *HRS* hepatorenal syndrome, *TIPS* transjugular intrahepatic portosystemic shunt, *LTx* liver transplantation, *ACLF* acute on chronic liver failure, *PPI* proton pump inhibitors, *CRP* C-reactive protein, *INR* international normalized Ratio.

### Incidence and clinical course of a nosocomial infection in male vs. female patients

**Figure 1:**
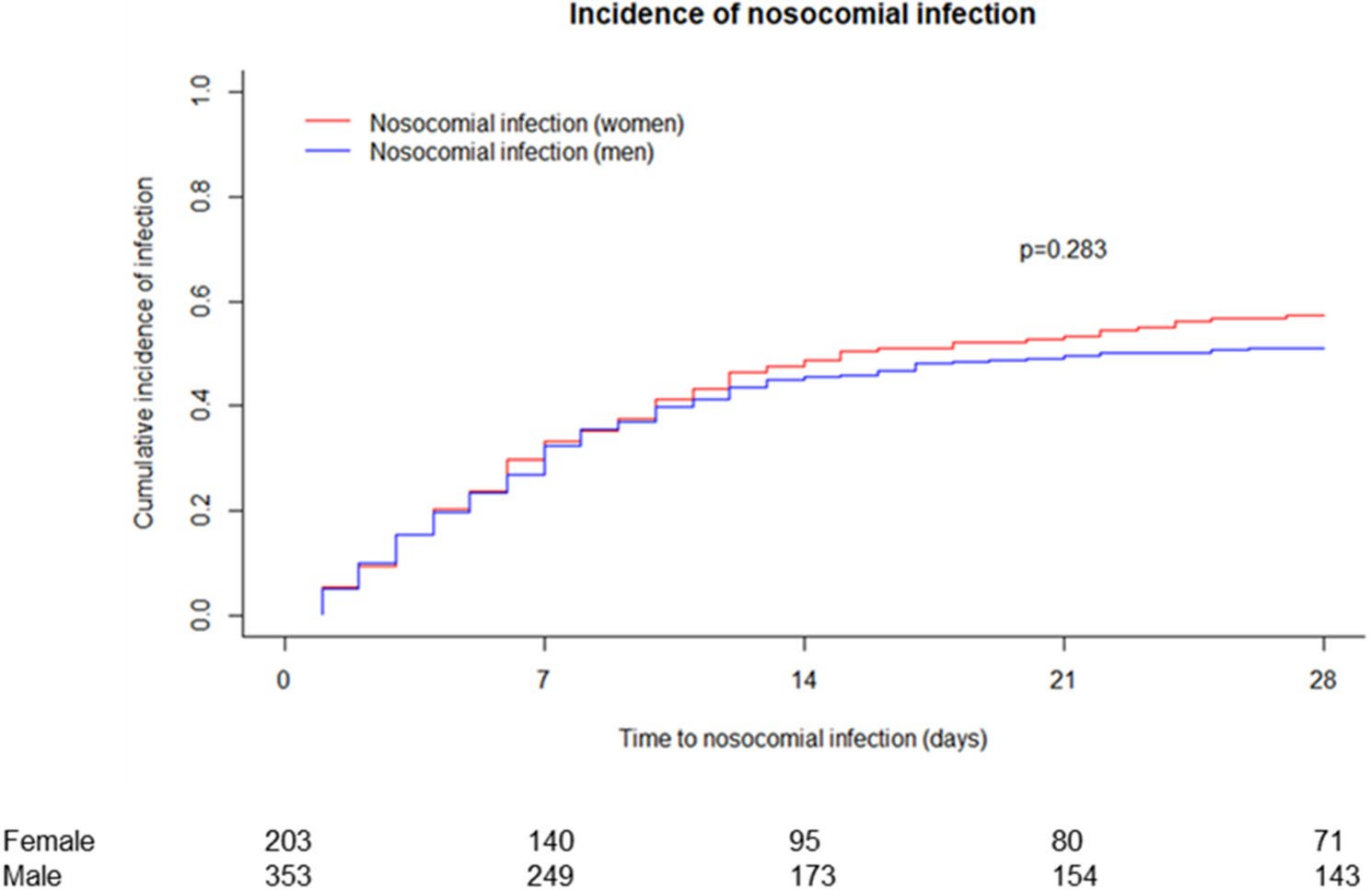
Incidence of nosocomial infection. Death and LTx were treated as competing risk. *LTx*: liver transplantation.

Overall, 258 patients (111 women and 174 men) developed a nosocomial infection. The cumulative incidence function indicated no major difference in the incidence of nosocomial infections between male and female patients (*P* = 0.283) (Fig. 1). Of note, after adjusting for other risk factors in the multivariate competing risk model, male sex was linked to a numerical lower hazard (HR: 0.81; *P* = 0.076). However, a statistically significant link to the incidence of nosocomial infections was only found for the MELD score (HR: 1.05; *P* < 0.001) (Table 2). Noteworthy, this was also consistent in both subgroups (female and male patients) and no sex-specific risk factors were detected.

**Table 2:** Multivariate competing risk analysis of incidence of nosocomial infection.

Variable (all patients)	*P*-Value	Hazard ratio (CI)
Male sex	0.076	0.8073 (0.6381, 1.0214)
MELD score	< 0.001	1.0515 (1.0333, 1.0700)
Platelets	0.12	1.0009 (0.9998, 1.0021)

Included parameters: sex, MELD score, diabetes, age and platelets.

*MELD* model of end stage liver disease, *CI* Confidence interval.

Outcome measures of nosocomial infection were AKI, ACLF and LTx-free survival. There was no difference between male and female patients in terms of the incidence of AKI, ACLF (data not shown) and LTx-free survival in the multivariate analysis (Table 5).

### Differences in the most common sites of nosocomial infections

**Figure 2:**
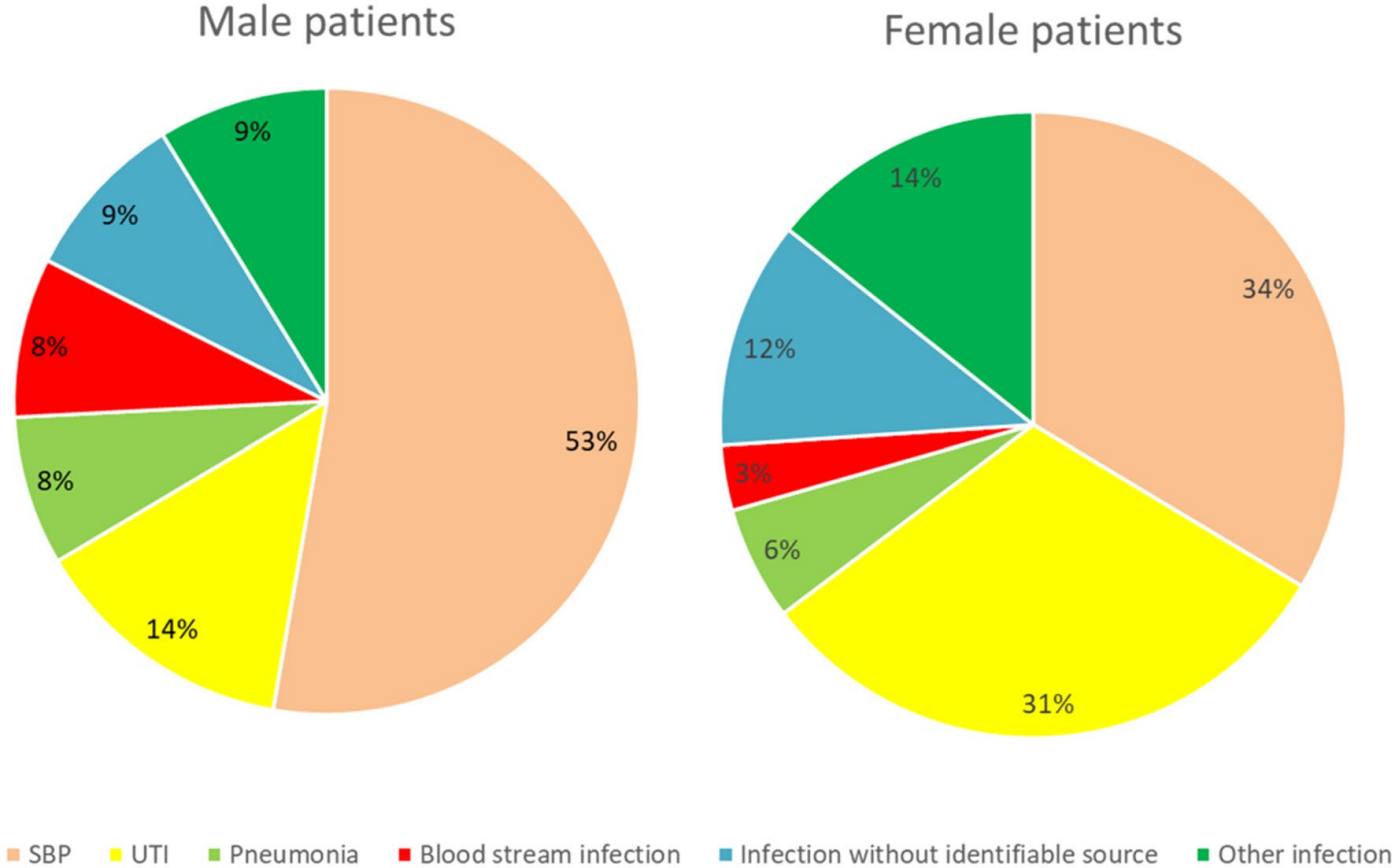
Distribution of site of nosocomial infection. In some patients more than one nosocomial infection was present. *SBP*: spontaneous bacterial peritonitis; *UTI*: urinary tract infection.

Overall, SBP (n = 136, 48%) was the most frequent nosocomial infection followed by UTI (n = 62, 22%). We documented only minor differences between male and female patients. SBP was the most frequent site of infection in men constituting to 53% (n = 96) of the infections. On the contrary, in only 34% (n = 40) of the infections in female patients a SBP was diagnosed (*P* = 0.048). In contrast, UTI was more common in females. In females 31% (n = 37) of the nosocomial infections could be attributed to the urinary tract whereas this was only the case in 14% (n = 25) of the infections in males (*P* < 0.001). Similar rates of pneumonia were detected among the infections in male and female patients (*P* = 0.758), while blood stream infections were more frequent in men (8% vs 3%, *P* = 0.154) (Fig. 2).

### Incidence and clinical course of as well as risk factor for nosocomial SBP in male vs. female patients

**Figure 3:**
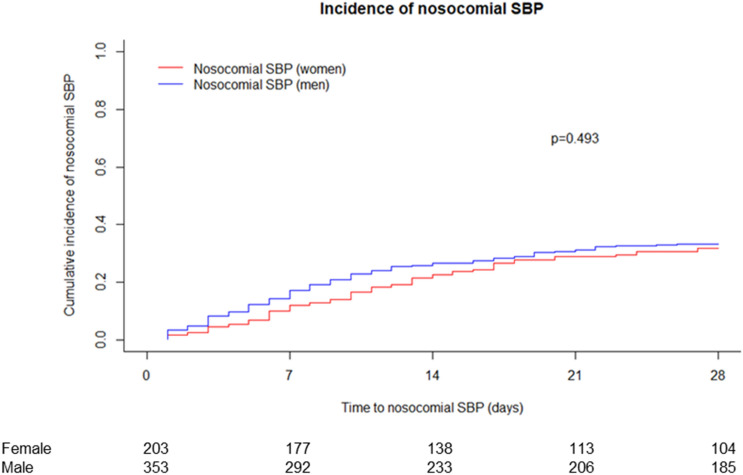
Incidence of nosocomial SBP. Death and LTx were treated as competing risk. *LTx* liver transplantation; *SBP* spontaneous bacterial peritonitis.

Although SBP was more frequent among the infection in males, the overall cumulative incidence of nosocomial SBP did not differ between male and female patients (*P* = 0.493) (Fig. 3). In the final multivariate competing risk model only presence of a peritoneal catheter (HR: 2.19; *P* < 0.001) was significantly linked to the development of nosocomial SBP (Table 3) in both female (HR: 3.46; *P* < 0.001) and male (HR: 1.85; *P* = 0.002) patients. In female patients the platelet count (HR: 1.003; *P* = 0.009) was also linked to SBP development.

**Table 3:** Multivariate competing risk analysis of incidence of nosocomial SBP.

Variable (all patients)	*P*-value	Hazard ratio (CI)
MELD score	0.088	1.0195 (0.9972, 1.0423)
Prior SBP	0.11	1.3445 (0.9337, 1.9359)
Platelets	0.074	1.0013 (0.9999, 1.0028)
Peritoneal catheter	< 0.001	2.1858 (1.5788, 3.0264)
Primary norfloxacin prophylaxis	0.076	0.5097 (0.2430, 1.0692)

Included parameters: sex, MELD score, diabetes, age, platelets, prior SBP, primary norfloxacin prophylaxis and peritoneal catheter.

*SBP* spontaneous bacterial peritonitis, *MELD* model of end stage liver disease, *CI* Confidence interval.

In 19 female patients (32%) with nosocomial SBP a pathogen was detected. In the majority of cases (84%) gram-positive bacteria were identified. Similarly, in 30 male patients with nosocomial SBP (27%) a pathogen was detected, which were also more frequently gram-positive bacteria (67%) (Suppl. Table 1).

The LTx-free survival after a nosocomial SBP did not differ between male and female patients (Table 6). Further, there was no difference between male and female patients in terms of the incidence of AKI and ACLF after nosocomial SBP (data not shown).

### Incidence and clinical course of nosocomial UTI in male vs. female patients

**Figure 4:**
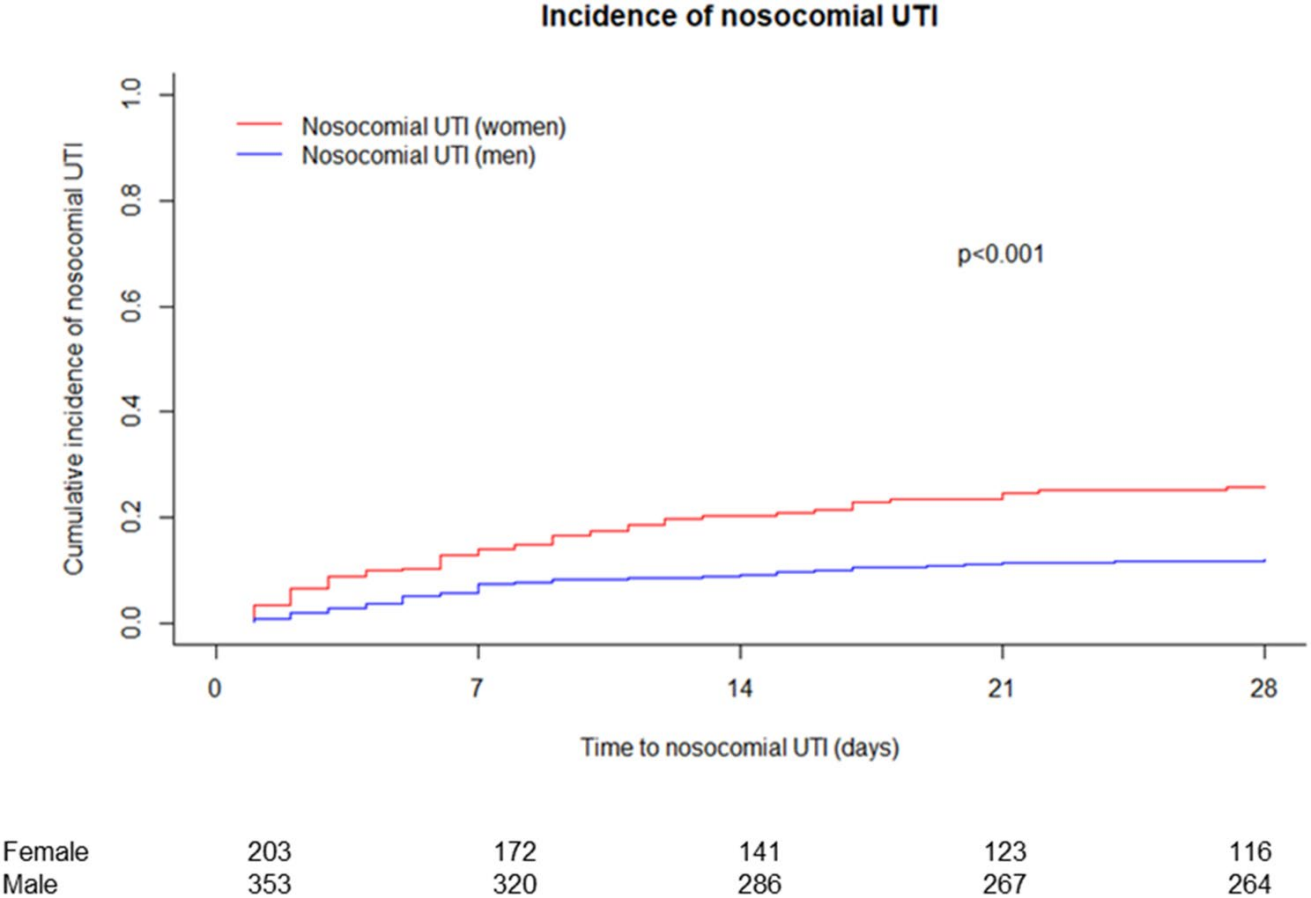
Incidence of nosocomial UTI. Death and LTx were treated as competing risk. *LTx* liver transplantation; *UTI* urinary tract infection.

Cumulative incidence of nosocomial UTI was significantly higher in female than in male patients (*P* < 0.001) (Fig. 4). Moreover, female sex was identified as an independent risk factor for the incidence of UTI in the multivariate competing risk analysis (HR: 2.16; *P* < 0.001). Other independent risk factors for UTI included use of a urinary catheter (HR: 3.94, *P* < 0.001) and a higher MELD score (HR: 1.04; *P* = 0.008) (Table 4). Of note, considering only male patients urinary catheter was detected as the only risk factor (HR: 5.4, *P* < 0.001).

**Table 4:** Multivariate competing risk analysis of incidence of nosocomial UTI.

Variable (all patients)	*P*-value	Hazard ratio (CI)
Sex (female)	< 0.001	2.1576 (1.3828, 3.3667)
Urinary catheter	< 0.001	3.9354 (2.4975, 6.2011)
MELD score	0.008	1.0386 (1.0099, 1.0681)
Age	0.053	1.0199 (0.9999, 1.0403)

Included parameters: Age, sex, urinary catheter, MELD score, diabetes and platelets.

*UTI* urinary tract infection, *MELD* model of end stage liver disease, *CI* Confidence interval.

Overall, in 24 female patients with nosocomial UTI a pathogen was detected (48%). Gram-positive bacteria and gram-negative bacteria were detected in 62% and 54%, respectively. In 11 female patients more than one pathogen was present. However, this was mostly due to the presence of Candida species. Further, in 24 of the male patients a pathogen was detected (59%). Gram-positive bacteria were identified in 16 male patients (67%) while gram-negative bacteria were present in 10 men (41%) and in 6 male patients more than one pathogen was detected (Suppl. Table 1).

Of note, among those with nosocomial UTI, sex was not documented as relevant risk factor for mortality/LTx (Table 7).

**Table 5:** Uni- and multivariate Cox-regression of LTx-free survival after the onset of nosocomial infection.

Variable	Univariate		Multivariate	
	*P*-value	Hazard ratio (CI)	*P*-value	Hazard ratio (CI)
Sex (male)	0.724	0.906 (0.522; 1.570)	0.614	
Age	0.420	0.991 (0.968; 1.014)		
Diabetes	0.763	0.908 (0.486; 1.698)		
Esophageal varices	0.705	0.891 (0.490; 1.620)		
β-Blocker	**0.003**	**0.333 (0.163; 0.683)**	**0.056**	**0.445 (0.194; 1.022)**
PPI	0.548	0.809 (0.405; 1.615)		
MELD score	** < 0.001**	**1.123 (1.081; 1.167)**	** < 0.001**	**1.109 (1.066; 1.152)**
Albumin	0.997	1.000 (0.938; 1.067)		
CRP	0.042	1.004 (1.000; 1.007)	0.073	
Leukocytes	0.067	1.017 (0.999; 1.036)		
Sodium	0.688	0.991 (0.950; 1.034)		
Platelets	0.046	0.966 (0.992; 1.000)	0.254	

Laboratory values were assessed at time of the onset of nosocomial infection.

Significant values are in bold.

*MELD* model of end stage liver disease, *CRP* C-reactive protein, *LTx* Liver transplantation, *PPI* proton pump inhibitors, CI Confidence interval.

**Table 6:** Uni- and multivariate Cox-regression of LTx-free survival after the onset of nosocomial SBP.

Variable	Univariate		Multivariate	
	*P*-value	Hazard ratio (CI)	*P*-value	Hazard ratio (CI)
Sex (male)	0.696	0.862 (0.410; 1.812)	0.659	
Age	0.682	1.007 (0.975; 1.039)		
Diabetes	0.360	0.659 (0.269; 1.611)		
Esophageal varices	0.735	0.870 (0.387; 1.954)		
β-Blocker	0.010	0.247 (0.086; 0.711)	0.058	0.349 (0.117; 1.038)
PPI	0.847	1.110 (0.385; 3.199)		
MELD score	** < 0.001**	**1.131 (1.082; 1.183)**	**0.001**	**1.096 (1.041; 1.154)**
Albumin	0.309	0.953 (0.869; 1.046)		
CRP	**0.001**	**1.006 (1.002; 1.009)**	** < 0.001**	**1.010 (1.005; 1.016)**
Leukocytes	0.077	1.018 (0.998; 1.038)		
Sodium	0.530	1.020 (0.960; 1.083)		
Platelets	0.013	0.992 (0.986; 0.998)	0.080	0.994 (0.988; 1.001)
Protein ascites	0.502	0.736 (0.300; 1.802)		
Nucleus containing cells	0.839	0.993 (0.932; 1.059)		

Laboratory values were assessed at time of the onset of nosocomial SBP.

Significant values are in bold.

*LTx* liver transplantation, *SBP* spontaneous bacterial peritonitis, *MELD* model of end stage liver disease, *CRP* C-reactive protein, *CI* Confidence interval.

**Table 7:** Uni- and multivariate Cox-regression of LTx-free survival after the onset of nosocomial UTI.

Variable	Univariate		Multivariate	
	*P*-value	Hazard ratio (CI)	*P*-value	Hazard ratio (CI)
Sex (male)	0.533	1.313 (0.557; 3.092)	0.769	
Age	0.851	1.004 (0.964; 1.045)		
Diabetes	0.560	1.349 (0.493; 3.693)		
Esophageal varices	0.166	2.372 (0.699; 8.056)		
β-Blocker	0.054	0.339 (0.133; 1.019)		
PPI	0.893	1.089 (0.315; 3.764)		
MELD score	** < 0.001**	**1.146 (1.085; 1.211)**	** < 0.001**	**1.146 (1.085; 1.211)**
Albumin	0.259	0.958 (0.889; 1.032)		
CRP	0.605	1.002 (0.995; 1.009)		
Leukocytes	0.058	1.060 (0.998; 1.127)		
Sodium	0.519	0.978 (0.914; 1.046)		
Platelets	0.223	0.996 (0.989; 1.002)		

Laboratory values were assessed at time of the onset of nosocomial UTI.

Significant values are in bold.

*LTx* liver transplantation, *UTI* urinary tract infection, *MELD* model of end stage liver disease, *CRP* C-reactive protein, *CI* Confidence interval.

## Discussion

There are significant differences in the etiology and the progression of liver disease between men and women^5–7^. Moreover, in those with end stage liver disease sex has been documented as a relevant factor with respect to the likelihood for transplantation and survival in some studies^14,15^. Nosocomial infections are a common and particularly severe complication at this stage that significantly alter the natural course of the disease^36^. Men and women differ in various parts including the distribution of immune cells^10^. Thus, we hypothesized that differences in the incidence and outcome of infections need to be considered that would partly explain the differences in the outcome of advanced liver cirrhosis that was documented between sexes in some studies. However, in our large well-defined cohort no major differences between male and female patients were identified.

There was a slightly, numerically lower incidence of nosocomial infections in male patients. However, this was not statistically significant after adjusting for the severity of liver disease and other relevant confounders. Moreover, the difference between males and females was mostly related to the higher incidence of UTI that was documented in female patients. The higher incidence of UTI is not surprising and well in line to other studies^37–40^. Of note, nosocomial UTI was not linked to a higher mortality compared to other infections. Thus, it seems unlikely that this results in a higher waiting list mortality that was described in prior studies^14,16,17,41^.

Of note, there was a trend towards a higher incidence of SBP in male patients. In line with our data O´Leary et al. reported a numerical higher incidence of SBP in men compared to women. However, this was not further investigated^16^. The main route of SBP is bacterial translocation from the intestinum^42^. Recent studies pointed out that the gut microbiome differs between men and women^43,44^, which may alter the likelihood for SBP as well as detectable bacteria. Indeed, there were some minor differences in the detected pathogens between male and female patients with SBP. However, the numbers were too small to draw any meaningful conclusion.

Infections are without a doubt one of the major hazards when managing female as well as male patients with end-stage liver disease. Early diagnosis, immediate treatment and in particular the development of prophylactic measures are key challenges to improve patients’ outcome. Therefore, it is important to define risk factors for infections in general as well as specific sites of infection in this cohort. We identified the presence of a peritoneal and urinary catheter as risk factor for the and development of nosocomial SBP and UTI, respectively. This was not different between male and female patients. Of note, in male patient urinary catheter was even the only independent risk factor for UTI. The high prevalence of UTIs in patients with urinary catheter is well in line with studies investigating the incidence of UTI in non-cirrhotic cohorts^45^. Temporary urinary catheter might be useful to document urinary output if required for patient management. The placement of a peritoneal catheter in hospitalized patients with ascites is not so common, so far. Nevertheless, repetitive paracentesis can be avoided^46^. However, our data emphasize that the indication for urinary catheter as well as peritoneal catheters should be critically evaluated and be removed as soon as possible^47^. This of particular importance in patients with decompensated cirrhosis who are particularly vulnerable for infections^48,49^.

The main limitation of our study is the retrospective single-center design. Therefore, some aspects of the management of patients might be center specific and the clinical diagnosis of infection may vary between the treating physicians. Moreover, the polymorphonuclear cell count in ascites was not available in our center at time of study inclusion. Therefore, in a few patients diagnosis of SBP might be a misclassification. However, the same criteria were applied in male and female patients. Further, a large number of patients was included in our study and our data provides some valuable information for future research questions. Moreover, it is to our best knowledge the first study that specifically addressed the impact of sex on the incidence and outcome of nosocomial infections among patients with decompensated liver cirrhosis.

To conclude, women developed only a numerical higher incidence of nosocomial infections, which are mostly due to a higher risk for UTI. Similar risk factors need to be considered for both sexes, which includes the use of temporary catheters. However, male and female do not differ with respect to the outcome of infections with respect to AKI, ACLF and LTx-free survival. Thus, a similar management seems appropriate.

## Supplementary Information

Supplementary Information.

## Abbreviations

LTx-free survival: Liver transplant free survival
AKI: Acute kidney injury
ACLF: Acute on chronic liver failure
UTI: Urinary tract infection
SBP: Spontaneous bacterial peritonitis
HIV: Human Immunodeficiency Virus
GFR: Glomerular filtration rate
EASL: The European Association for the Study of the Liver
PPI: Proton pump inhibitors
CRP: C-reactive protein
AIC: Akaike’s information criterion
HR: Hazard Ratio
CI: Confidence interval
HE: Hepatic encephalopathy
HRS: Hepatorenal syndrome
TIPS: Transjugular intrahepatic portosystemic shunt
LTx: Liver transplantation
