# Supplementary material for: Nosocomial infections in female compared with male patients with decompensated liver cirrhosis
Source: Sci Rep. 2022 Feb 28;12:3285. doi: 10.1038/s41598-022-07084-9 (PMC8885665; doi:10.1038/s41598-022-07084-9)
Supplement: Supplementary file 1 — Supplementary Information. [file 41598_2022_7084_MOESM1_ESM.docx]

**Supplementary Figure 1:** Flowchart of patient recruitment


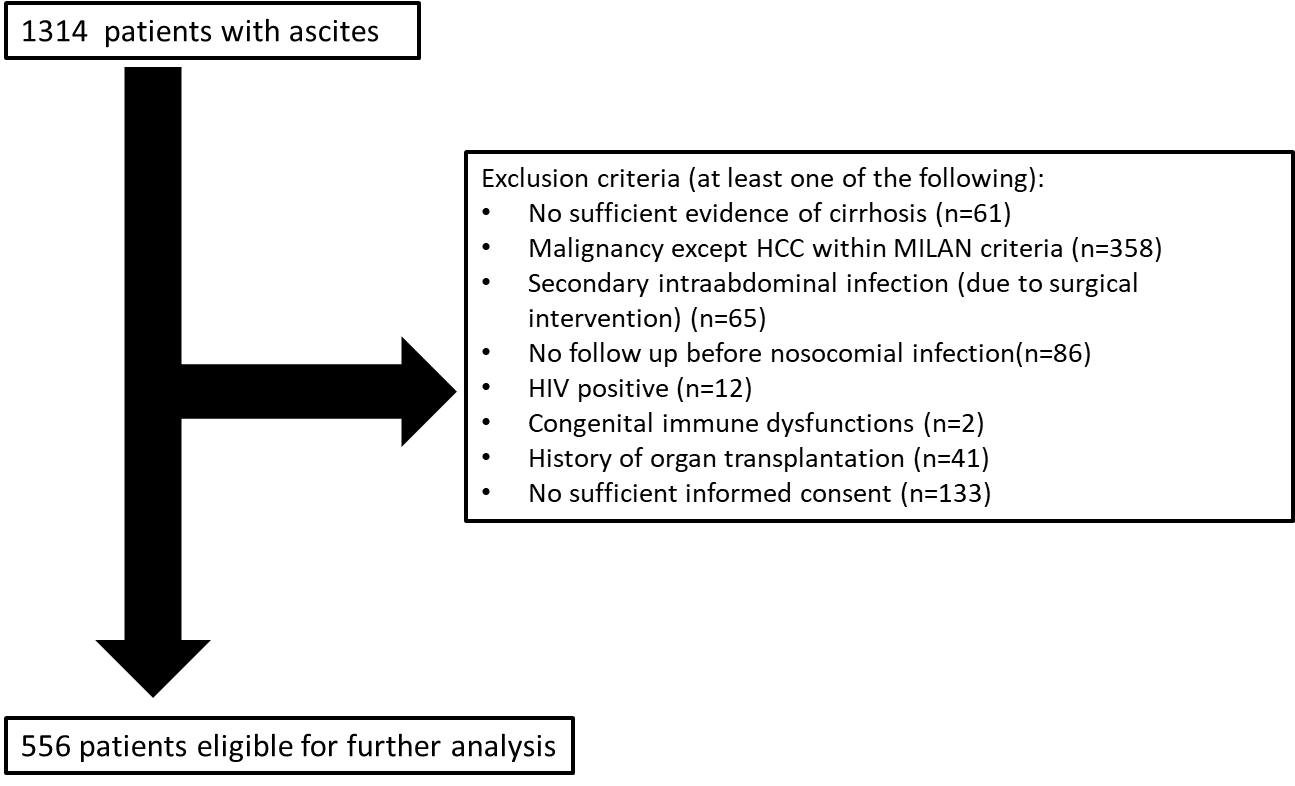


Abbreviations: HCC: hepatocellular carcinoma; HIV: Human Immunodeficiency Virus

**Supplementary Table 1:** Detected pathogens at time of SBP and UTI

| SBP | UTI |
| --- | --- |
| Acinetobacter lwoffii | Acinetobacter sp |
| Acromobacter xylosidans | Candida albicans |
| Bacillus sp., Paenibacillus sp. | Candida albicans/dubliensis |
| Corynebacterium amycolatum | Candida glabrata |
| Corynebacterium amycolatum /xerosis | Candida kefyr |
| Corynebacterium jeikeium | Candida krusei |
| Corynebacterium minutissium | Candida sp. |
| E. coli | Candida tropicalis |
| E. coli (3 MRGN) | Citrobacter freundii |
| Enterobacter cloacae | E. coli |
| Enterococcus casseliflavus | E. coli (3 MRGN) |
| Enterococcus cecorum | Enterococcus faecalis |
| Enterococcus faecium | Enterococcus faecalis (VRE) |
| Enterocuccus faecalis | Enterococcus faecium |
| Klebsiella pneumoniae | Enterococcus faecium (VRE) |
| Listeria monocytogenes | Enterobacter cloacae |
| Staphylococcus xylosus | Enterobacter cloacae (3MRGN) |
| Staphylococcus aureus | Klebsiella oxytoca |
| Staphylococcus aureus (MRSA) | Klebsiella pneumoniae |
| Staphylococcus auricularis | Lactococcus lactis |
| Staphylococcus epidermidis | Pseudomonas aeruginosa |
| Staphylococcus haemolyticus | Sphingomonas paucimobilis |
| Staphylococcus hominis | Staphylococcus epidermidis |
| Streptococcus agalactiae | Staphylococcus aureus |
| Streptococcus parasanguinis | Staphylococcus aureus (MRSA) |
| Streptococcus sanguinis | Staphylococcus hominis |
| Streptococcus mitis | Streptococcus agalactiae |
| Streptococcus oralis | Streptococcus viridans |
| Streptocoocus vestibularis |  |
